# Dizziness and vertigo sick leave before and after insurance restrictions – a descriptive Swedish nationwide register linkage study

**DOI:** 10.1186/s12889-024-20119-2

**Published:** 2024-09-27

**Authors:** Katarina Zborayova, Marie-Louise Barrenäs, Gabriel Granåsen, Kevin Kerber, Jonatan Salzer

**Affiliations:** 1 Department of Clinical Sciences, Otorhinolaryngology, Umeå University, Umeå, Sweden; 2 Department of Public Health and Clinical Medicine, Umeå University, Umeå, Sweden; 3 Department of Neurology, Ohio State University, Columbus, OH USA; 4 Department of Clinical Sciences, Neurosciences, Umeå University, Umeå, Sweden

**Keywords:** Sick leave/sickness absence, Vertigo, Dizziness

## Abstract

**Background:**

Vertigo and dizziness can be disabling symptoms that result in sick leave. Research regarding sickness absence due to dizziness has focused on specific vestibular diagnoses rather than the nonspecific vertigo/dizziness diagnoses. Strict sick leave regulations were introduced in Sweden in 2008. The aim of this study was to describe the vertigo/dizziness sick leave prevalence and duration considering both specific and nonspecific diagnoses according to International Classification of diseases 10th revision (ICD-10) on the 3-digit level, including the less specific “R” diagnoses.

**Methods:**

Through Swedish nationwide registers we identified individuals aged 16–64 years who during the years 2005–2018 were sickness absent > 14 consecutive days – minimum register threshold – due to vertigo/dizziness diagnoses according to ICD10 codes: specific diagnoses (H81.0, H81.1, H81.2, H81.3, H81.4, G11x) and nonspecific (R42, R26, R27, H81.9). We described the demographic characteristics, prevalence and duration of such sick-leave spells. Data were stratified according to diagnostic groups: ataxias, vestibular and nonspecific.

**Results:**

We identified 52,179 dizziness/vertigo sick leave episodes > 14 days in 45,353 unique individuals between 2005–2018, which constitutes 0.83% from all sick leave episodes in the given period.The nonspecific diagnoses represented 72% (*n* = 37741) of sick leave episodes and specific vestibular H-diagnoses 27% (*n* = 14083). The most common specific vestibular codes was Benign paroxysmal positional vertigo (BPPV) 9.4% (*n* = 4929). The median duration of sick leave was 31 days (IQR 21–61). Women on sick leave were younger than men (47 vs 51 years, *p* < 0.05) and had a higher proportion of nonspecific diagnoses compared with men (74% vs 70%, *p* < 0.05).

**Conclusions:**

The vast majority of vertigo/dizziness sick leave episodes were coded as nonspecific diagnoses and occurred in women. BPPV, a curable vestibular condition, was the most common specific diagnosis. This suggests a potential for improved diagnostics. Women on sick leave due to dizziness/vertigo were younger and more often received nonspecific diagnostic codes. Future studies should determine the frequency of use of evidence based therapies and investigate further the gender differences.

## Background

Vertigo and dizziness are common and can be disabling symptoms that result in sick leave. They lead to health care utilization and constitute economic and social burden [1–3]. In Sweden, sick leave due to dizziness/vertigo constitutes 0.83% of all sick leaves [4]. Individuals with dizziness may experience reduced balance, increased risk of falls and anxiety due to fear of a serious illness, making it challenging to carry out daily activities and maintain employment [2, 5–7]. There are some highly effective evidence-based treatments for specific causes, but they are frequently underused.  In recent years, a growing interest in understanding not only etiology but also the impact of sickness absence due to dizziness has arisen. Dizziness is not a disease, but a leading symptom of various conditions with different causes, including the inner ear, brainstem, cerebellum, or psychological factors. Prior studies about dizziness and sick leave have focused on specific vestibular “H” diagnoses (e.g., H81.x) rather than the nonspecific “R” diagnoses (e.g., R42) as defined by the ICD-10 system, where dizziness is diagnosed only as a symptom and not a disorder [1]. In 2008, due to escalating sick leave-related costs for mental health, the Swedish regulations for sick leave compensation were changed and restricted to one year in most cases [8]. After this shift in labour market insurance politics an overall reduction in sick leave was observed [9].

Prior studies on dizziness etiology in primary care have found that up to 80% of cases were assigned nonspecific diagnoses, with a large variation between studies [10]. Furthermore, Benecke et al has shown that one third of patients with peripheral vestibular vertigo, who were classified as “normal, not at all ill” by their physicians using the Clinical Global Impression score, had lost working days [11, 12].

A few cross-sectional studies have investigated sick leave due to dizziness/vertigo. A national Norwegian study reported that 0.9% of women and 0.7% of men on ≥8 weeks of sick leave were on sick leave due to dizziness/vertigo [13], and a German study reported that 53% of dizziness/vertigo sickness absentees were on sick leave due to non-vestibular causes [2]. Two Swedish investigations have been performed; one regional study found that 82% of patients on sick leave due to otoaudiological diagnoses were certified with vertigo without hearing symptoms [14], and a more recent national investigation confirmed that vestibular disorders were the most common reason for audio-vestibular sick-leave [15, 16]. These studies are all lacking analyses of sick leave over time and stratification for specific ICD-10 codes for the vestibular disorders Benign paroxysmal positional vertigo (BPPV), vestibular neuronitis and Meniere’s disease, as well as nonspecific symptom diagnoses.

The lifetime prevalence of BPPV is 2.4% and the condition is treatable with a simple repositioning maneuver in the majority of cases [17, 18]. BPPV appears to be underdiagnosed as a cause of dizziness/vertigo among elderly patients in primary health care [19]. Sick leave due to BPPV has been little studied but a recent Mexican study revealed a high proportion of BPPV among dizziness sick leave certificates [20].

The aim of this Swedish nationwide study was to investigate and report the dizziness and vertigo sick leave prevalence, durations and volumes stratified by diagnoses according to the International Classification of diseases 10^th^ revision (ICD-10) on the 3-digit level, including the nonspecific “R” diagnoses. The monitored period (2005–2018) covers time both before and after the sick leave reimbursement policy shift in 2008, which offers this unique opportunity to report the impact from restricted sick leave compensation regulations. The results of this study may be relevant to politicians, employers, and healthcare professionals working to reduce the impact of sickness absence on the Swedish population.

## Methods

### The Swedish sickness insurance system in brief

All individuals in Sweden with income from work or unemployment benefits are covered by the same public sickness insurance, providing sick leave benefits to people who due to disease cannot work. Sick leave during the first seven days – including one qualifying day without economic reimbursement – is, with some exceptions, self-certified. A sickness certificate issued by a physician is required from the 8^th^ day of sick leave spell. Sickness benefits are paid by the Social Insurance Agency from day 15 (inclusive); the first 14 days of sick leave is usually paid by the employer. All physicians, regardless of medical specialty, may issue a sickness benefit certificate. The certificate specifies the patient’s diagnosis (ICD-10 code), assessment of the patient’s functional impairment and limitation of activities and recommended length and degree (100%, 75%, 50% or 25% of full-time) of sick leave. The tool used to follow volumes of sick leave over time in Sweden is the Sickness Cash Benefit Rate, defined below.

All sick leave episodes with dizziness/vertigo ICD-10 as the primary diagnostic code that started between January 1^st^ 2005 and December 31^st^, 2018, for persons aged 16–64, not on disability or old age pension, were identified in the LISA database (Longitudinell Integrationsdatabas för Sjukförsäkrings- och Arbetsmarknadsstudier) at the Swedish Social Insurance Agency (Försäkringskassan). The LISA database is population-based and contains data on all sick leave spells >14 days and unemployment for all Swedish citizens aged ≥16 years of age including diagnoses. Diagnostic codes were registered on letter + 3-digit level (G11.x; H81.x, R26.x, R27.x; R42). The dizziness/vertigo-diagnoses were categorized into three groups: *Ataxias* (G11.0, G11.1, G11.2, G11.4, G11.8); *Vestibular diseases* (Meniere’s disease (H81.0), benign paroxysmal positional vertigo (H81.1), vestibular neuronitis (H81.2), other peripheral vertigo (H81.3), vertigo of central cause (H81.4)); and *Nonspecific symptom diagnoses* including dizziness and vertigo not otherwise specified (R42), abnormalities of gait and mobility (R26x), other lack of coordination (R27x), and unspecified disorder of vestibular function (H81.9).

Each individual was matched using the social security numbers to a database at Statistic Sweden to add data on educational and income level.

### Statistical analysis

The cohort characteristics and distribution of sick leave spells over ICD-10 diagnoses are displayed using descriptive statistics. The sickness cash benefit rate, i.e. the number of full (100%) sick days per eligible person (Swedish inhabitants aged 16–64, not on old age or disability pension) and year; and the number of sick leave days (duration) by year per eligible inhabitant were calculated.

For the estimation of sick leave *duration* we decided to exclude the last three years of the study period (2016-2018) to avoid too short duration estimations. For the estimation of sickness cash benefit rate (*volumes*) per year we excluded the first three years (2005-2007) after observing that the volumes over time indicated that sick leave spells initiated during the years before 2005 were not included in the data extraction.

The chi-square test was used to test for differences between proportions.

## Results

### A. Episodes

The LISA database contained 6308950 sick leave episodes between 2005–2018 and 52179 (0.83%) of these had dizziness/vertigo as the primary reason [4].

Demographic characteristics of 52179 sick-leave episodes due to dizziness/vertigo that started between 2005–2018, stratified by ICD-10 categories, are presented in Table 1.

**Table 1:** Characteristics of all sick leave episodes > 14 days due to dizziness/vertigo in Sweden between 2005–2018 stratified by ICD-10 diagnostic groups

Characteristic	Overall	Ataxias	Vestibular	Nonspecific
	*N* = 52179	*N* = 355	*N* = 14083	*N* = 37741
Age (years)	49 (39–57)	49 (40–57)	51 (42–58)	48 (38–57)
Men	17297 (33%)	170 (48%)	5042 (36%)	12085 (32%)
Women	34882 (67%)	185 (52%)	9041 (64%)	25656 (68%)
Median (IQR) net sickleave days^a^	31 (21–61)	274 (86–582)	29 (21–53)	31 (21–63)
Mean (SD) degree of sickleave (proportions of full days, percentages)^b^	87% (21%)	79% (27%)	87% (21%)	87% (21%)

Data are median (interquartile range), n (%) or mean (standard deviation)

^a^As the Social Insurance Agency covers sickness benefits from day 15, only episodes > 14 days long are registered in the LISA database

^b^It is possible to be on sick leave for full days (100%) or part of days (75%, 50%, 25%). Lower degrees are used when shorter days, or fewer days per week, are necessary for recovery. It is the issuing physician who recommends degree of sick leave in the certificate

The nonspecific diagnoses constituted the bulk of sick-leave episodes, 72% (*n* = 37741) while vestibular H-diagnoses caused 27% (*n* = 14083) of episodes and the ataxias only 1% (*n* = 355; Table 1). There were twice as many women as compared to men on sick leave due to dizziness/vertigo in Sweden during the studied years. The sick leave episodes due to ataxias had a longer median duration compared with those with vestibular and nonspecific causes (Table 1). The overall mean duration was 17 days in addition to the 14 days paid by the employer, giving on average one month sick leave per episode. The median (IQR, min–max) total sick leave duration was 31 days (21–61, 14.25–3182); 1% of episodes were longer than 3 years.

**Fig. 1:**
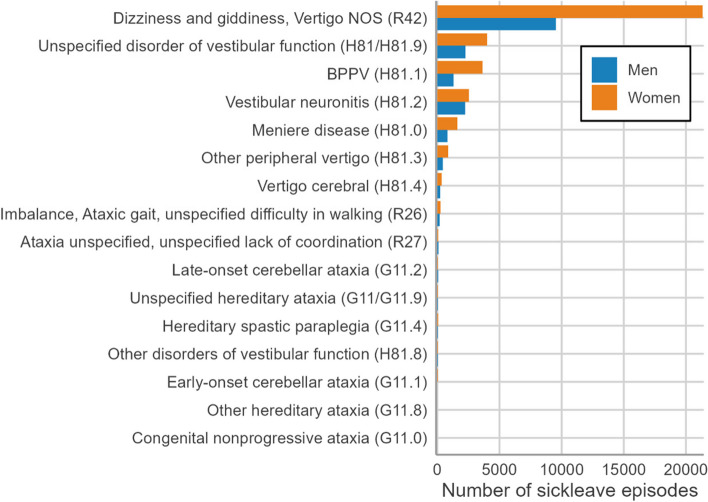
Total number of sick leave episodes > 14 days due to dizziness/vertigo in Sweden between 2005–2018 according to ICD-10 diagnoses

As shown in Fig. 1 two nonspecific ICD-10 codes (R42 and H81.9) represented the two largest groups, 59% and 12% of the total number of sick leave spells; and BPPV (H81.1) was the third most common diagnosis – and the most common specific diagnosis – constituting 9.4% of all sick leave episodes. The women:men ratios for sick leave episodes were 2.02 for the entire cohort and 2.12 for the nonspecific diagnoses.

In total, 45353 unique individuals were included. The majority (*n* = 40288, 89%) had experienced one sick leave spell and the largest number of sick leave spells experienced by one person was 14 (Table 2).

**Table 2:** Number of sick leave episodes with a duration of > 14 days due to dizziness/vertigo per subject between 2005–2018

Number of episodes	Number of subjects
1	40288 (88.8%)
2	3981 (8.8%)
3	717 (1.6%)
4	219 (0.54%)
≥ 5	147 (0.36%)
Total	45353 (100%)

Table 3 summarizes the demographic characteristics of the 45353 individuals stratified by age, education, income and gender. Sick leave due dizziness/vertigo was more common among women with either a low income or high education compared with the corresponding male groups (*p* < 0.05 for both comparisons). Each subject were assigned an income group quartile according to Statistics Sweden’s population data where the first quartile is considered low income and the fourth quartile high income. The second and third quartile (normal income) were merged.

**Table 3:** Characteristics of all individuals on sick leave > 14 days due to dizziness/vertigo in Sweden between 2005–2018 stratified by gender

Characteristic	Men				Women			
	**Overall**	**Ataxias**	**Vestibular**	**Nonspecific**	**Overall**	**Ataxias**	**Vestibular**	**Nonspecific**
	*N* = 15152	*N* = 130	*N* = 4235	*N* = 10787	*N* = 30201	*N* = 132	*N* = 7396	*N* = 22673
Age^a^ (years)	51 (41–58)	52 (44–58)	51 (42–59)	51 (40–58)	47 (37–56)	48 (38–57)	49 (40–57)	46 (36–56)
Age^b^ (groups)								
18–30	1354 (8.9%)	8 (6.2%)	315 (7.4%)	1031 (9.6%)	3609 (12%)	15 (11%)	600 (8.1%)	2994 (13%)
31–40	2291 (15%)	18 (14%)	570 (13%)	1703 (16%)	6252 (21%)	23 (17%)	1317 (18%)	4912 (22%)
41–50	3765 (25%)	34 (26%)	1114 (26%)	2617 (24%)	8087 (27%)	32 (24%)	2084 (28%)	5971 (26%)
51–60	5232 (35%)	52 (40%)	1523 (36%)	3657 (34%)	8714 (29%)	47 (36%)	2388 (32%)	6279 (28%)
61–65	2333 (15%)	18 (14%)	649 (15%)	1666 (15%)	3374 (11%)	15 (11%)	950 (13%)	2409 (11%)
65 +	177 (1.2%)	0 (0%)	64 (1.5%)	113 (1.0%)	165 (0.5%)	0 (0%)	57 (0.8%)	108 (0.5%)
Education^b^ (years)								
0–9	3205 (**21%**)	25 (19%)	771 (18%)	2409 (22%)	3429 (**11%**)	10 (7.6%)	696 (9.4%)	2723 (12%)
10–12	8440 (56%)	70 (54%)	2378 (56%)	5992 (56%)	15,078 (50%)	66 (50%)	3563 (48%)	11449 (51%)
≥ 12	3477 (**23%**)	35 (27%)	1079 (26%)	2363 (22%)	11,662 (**39%** **)**	56 (42%)	3129 (42%)	8477 (37%)
Unknown	30	0	7	23	32	0	8	24
Income level, quartiles^b^								
< First quartile	2048 (**14%**)	39 (30%)	375 (9%)	1634 (15%)	8661 (**29%**)	64 (48%)	1728 (23%)	6869 (30%)
Interquartile range	7460 (49%)	57 (44%)	2030 (48%)	5373 (50%)	15450 (51%)	49 (37%)	3955 (54%)	11446 (51%)
> Forth quartile	5601 (**37%**)	34 (26%)	1817 (43%)	3750 (35%)	5977 (**20%**)	19 (14%)	1688 (23%)	4270 (19%)
Unknown	43	0	13	30	113	0	25	88
Net sick leave days^a^	32 (21–63)	314 (91–633)	29 (21–50)	33 (21–68)	29 (21–54)	334 (105–586)	28 (20–46)	30 (21–57)

^a^Median (25%–75%)

^b^n (%)

### B. Sickness cash benefit rate, sex and social factors

**Fig. 2:**
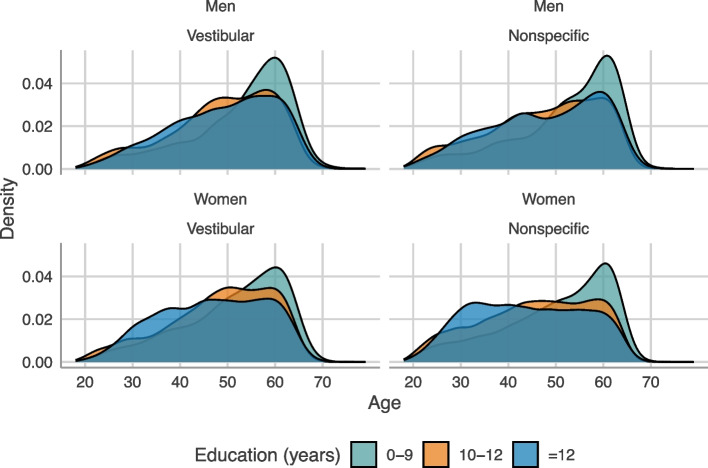
Distribution of age at sick leave episode start due to dizziness/vertigo in Sweden between 2005–2018 stratified by diagnostics groups, gender and education

As shown in Fig. 2, the age distribution at the start of the sick leave episodes seems associated with sex and education level. While there is a steady increase of sick leave with increasing age among men regardless of education level, more women with the highest education (> 12 years) go on sick leave already in the 4th decade of life.

**Fig. 3:**
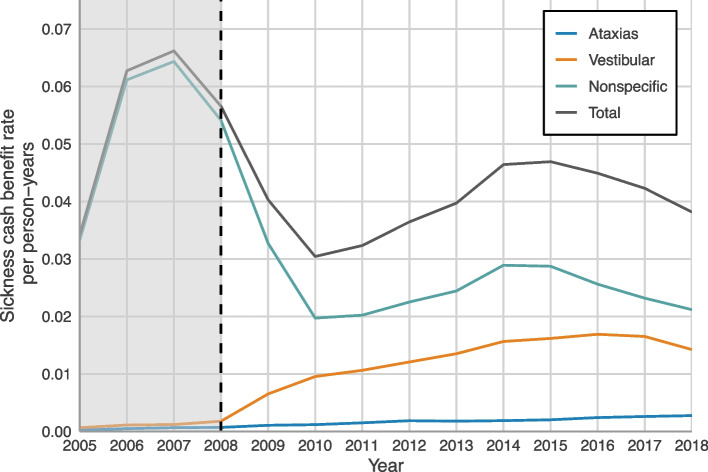
Sickness cash benefit rate of sick leave > 14 days due to dizziness and vertigo in Sweden between 2005–2018 stratified by diagnosis group

Figure 3 shows the Sickness Cash Benefit Rate, i.e. the number of full (100%) sick days per eligible person (Swedish inhabitants aged 16–64, not on old age or disability pension) and year, between 2005 and 2018. As illustrated, the highest rates were seen for nonspecific diagnoses, with an escalation during 2005–2007 and a decline during the 2008–2010 period*.* After this a less pronounced rise in rates with a peak around 2014–2015 were observed. The rates from vestibular disease and ataxias show a slow and more steady increase over time during the 13-years period.

## Discussion

### Summary of principal findings

This Swedish population-based register study has reported on 52179 sick leave episodes longer than 14 days among 45353 individuals due to dizziness and vertigo during the years 2005–2018, i.e. 0.83% of all sick leave episodes in Sweden during the period. The most common ICD-10 diagnosis category was nonspecific symptom diagnoses (72%), followed by specific vestibular disorders (27%) and ataxias (1%), the latter having the longest sick leave durations. Benign paroxysmal positional vertigo was the most common specific diagnosis (9.4% (*n* = 4929)). As previously shown, more women than men were on sick leave due to all dizziness/vertigo diagnoses, apart from vestibular neuronitis and ataxias. The longitudinal population-based approach entails a high degree of generalizability within Sweden and other countries with similar health insurance systems. The inclusion of nonspecific dizziness diagnostic codes and 3-digit ICD-10 codes is unique and adds important dimensions in the understanding of work capacity reduction caused by dizziness.

### Nonspecific dizziness/vertigo sick leave episodes

The proportion of nonspecific diagnoses in previous studies on dizziness sick leave range from 0.0–80% [2, 10, 13]. The differences between studies may be explained by different study designs. The Norwegian study used register data from 1997 after eight weeks of sick leave and the International Classification of Primary Care (ICPC), while the German study population of 1003 participants were selected from the German National Health Interview Survey (*n* = 8318; response rate 52% in the first step) using a validated neurotologic interview to separate vestibular vertigo from non-vestibular dizziness. The high proportion in the current Swedish study might reflect differences relating to attitudes or diagnostic efforts between countries.

Systematic reviews on the burden of dizziness in primary care [10] report that 13 out of 14 studies used a nonspecific diagnostic category and up to 80% of cases were assigned to that category [1, 21]. This is worrying, at least from a Swedish point of view, as the proportion of sick absentees due to nonspecific dizziness/vertigo diagnoses in the current study is high. Although nonspecific dizziness/vertigo appears to entail a low risk for transition into disability pension [14], our study confirms that nonspecific dizziness/vertigo diagnosis is an important contributing factor to the total sick leave volume, implicating a need further research into causes and prevention. In particular, participation in treatment for the suspected underlying cause and/or general vestibular rehabilitation should be given high priority in future intervention programs.

### Nonspecific dizziness/vertigo and Sickness Cash Benefit Rate

In our study, by describing the annual Sickness Cash Benefit Rate, it was possible to observe changes during and after the period 2005–2008, when the Swedish sick leave regulations became more restricted. Before 2008, there was no determined official time limit in Sweden for how long a person could receive sickness cash benefit. However, since 2008, the compensation has been restricted to one year in most instances. The results show that these time limits and the diagnoses-specific sick listing guidelines introduced in 2008 were associated with a reduction in the prevalence of sick leave spells for nonspecific dizziness (Fig. 3) as for most other diagnosis [9, 22, 23], but not for vestibular nor ataxia diagnoses. The reason for this may be that policy changes have a greater impact on sick leave due to nonspecific symptoms than they do on sick leave due to diagnoses with clear diagnostic criteria and specific clinical findings, such as nystagmus and reduced vestibulo-ocular reflex.

Sick leave is an outcome variable that represents several complex interactions between work-related, domestic and personal factors or stressors and morbidity as argued by Marmot et al. [24]. The concepts of Chronic Subjective Dizziness (CSD), Phobic Postural Vertigo (PPV) and Persistent Postural Perceptive Dizziness (PPPD) all updates the psychosomatic-somatopsychic dyad providing insights into the events triggering and sustaining self-perpetuating subjective chronic dizziness, that is not due to an active vestibular deficit [25–28]. Still, despite clear correlations between vestibular symptoms and psychiatric morbidity, panic disorder and major depression being the commonest psychiatric diagnoses, Sickness Cash Benefit Rate for the vestibular diagnoses did not decline after the policy change as did nonspecific diagnoses. This difference suggests that the diagnostic procedures when prescribing sick leave for patients with dizziness need to improve.

We see at least four possible scenarios explaining the nonspecific diagnoses being overused on the sick leave certificates.

1. The exact diagnosis is unknown, either due to insufficient clinical examination or due to difficulties determining an etiologic diagnosis despite meticulous investigation.
2. Psychiatric comorbidities or exhaustion conditions with vertigo/dizziness as one of the symptoms are coded as R42. Perhaps issues like stigma may incline patient and physician to choose symptom descriptions leaning toward somatic disease rather than disclosing psychiatric illness.
3. The patient suffers a kind of chronic dizziness form PPV/CSD or vestibular migraine and the physician use a diagnoses R42 because neither PPV,CSD, nor vestibular migraine have their exclusive codes in ICD-10 classification.
4. The treating provider has actually made a more specific diagnosis but used a nonspecific code in the primary position, and the more specific code in the secondary position or not at all.

It is also possible, that the sick leave episodes became shorter than 15 days after 2008 and therefore were not detected in this register-based study as short sick leave episodes are paid by the employer and not registered.

Predictors of dizziness in the general population include female sex, high age, and low education [5, 13, 29–31]. The current study, when stratified for gender, age and ICD-10 category, did not fully confirm previous studies as the risk for sick leave due to nonspecific and vestibular dizziness/vertigo appeared unrelated to age among highly educated women. The spells peaked already in the 4th decade of life and stayed independent of age. The earlier rise among highly educated women is similar to the burnout sick leaves in highly educated women seen by the Finnish Institute of Occupational Health, possibly hinting towards similar processes and suggesting that dizziness may be used in lieu of mental health disorders [32].

Recently, it was found that women have an unjustified 14 more days in sick leave due to mild or moderate mental ill health compared to men, despite the same level of assessed work capacity and self-assessed health [33]. The physician´s assessment of the need for sick leave could explain 13,7 out of the 14 days. The difference was particularly apparent among women aged 31 to 40, without children, with higher education, who had high income and who predominately worked in male-dominated sectors. It is possible that similar gender-biased differential assessments of work capacity, in the context dizziness, may explain the higher frequency of sick leave among women, as well as the high Sickness Cash Benefit Rate due to nonspecific symptom diagnoses in the current study. This warrants further research.

### Vestibular sick leave episodes and ataxias

The vestibular sick leave cases constituted 27% of the total sample and among the specific vestibular disorders, BPPV constituted the largest group. This is, at least to our knowledge, the first report on vestibular sick leave at 3-digit ICD-level. The median net sick leave days were 29 days in the vestibular group and 31 days in the nonspecific group, i.e. similar duration. Studies on the prevalence of vestibular vertigo in primary care show large variations, BPPV ranging from 4% to 40% and vestibular neuronitis from 1% to 24% [10]. A German study shows that vestibular vertigo compared with non-vestibular dizziness was more frequently followed by sick leave (41% vs 15%; *P*<0.001) [2]. The findings regarding permanently reduced work capacity from vestibular disorders demanding sick leave are conflicting: A multi-country study on the impact of vestibular disorders on work productivity showed that 70% of the patients in employment had reduced their workload, 63% had lost working days due to vertigo, 4.6% had changed their job and 5.7% had quit work completely due to vertigo [11]. Skoien et al., reported that disability pension was granted within 5 years for 29% of women and 23% of men who had been registered for an eight week long sick leave period due to a vestibular diagnosis [13]. In a Swedish study however, no association was observed between sick leave due to vertigo diagnoses and disability pension [16].

Although repositioning maneuvers and vestibular rehabilitation to reduce dizziness and vertigo symptoms are evidence-based and relatively easy to perform, studies have repeatedly shown these to be substantially underused [19, 34–36]. In the present study, BPPV diagnosis constituted a major contributor to the vestibular vertigo sick leave episodes ≥14 days long – again suggesting underusage of potentially curing repositioning maneuvers which a systematic review found are effective in ~56% of persons with posterior canal benign positional paroxysmal vertigo after a single treatment [17]. Similarly, vestibular rehabilitation is an evidence-based treatment for chronic dizziness but is used in <5% of target patients in primary care [37–39]. Clinical practice guidelines worldwide state that such rehabilitation should be used and it is possible that the rates of sick leave based on nonspecific dizziness may also be reduced by vestibular rehabilitation [40–45].

The number of persons on sick leave due to ataxias in this study were too few for any conclusions to be drawn.

### Limitations

This observational study design provides hypotheses-generating data but does not allow us to draw conclusions on causality. Sick leave episodes shorter than 15 days were not included in the study as these are not covered by the Swedish compulsory sickness insurance, and therefore not registered at the Swedish Social Insurance Agency. This, together with the truncation of years in the estimations of Sickness Cash Benefit Rate, leads to an underestimation of the true volumes of sick leave due to dizziness and vertigo. Due to the data being delivered without personal identifiers no diagnostic code validation was possible. Another limitation of the study is the lack of a control group, e.g. dizzy persons not on sick leave, and the inability to account for comorbidity, notably psychiatric comorbidity, which has been shown to increase sick leave duration [7, 46]. Also, psychosocial workplace characteristics that might affect the well-being of employees and the lengths of sick leave were not considered in this study [47, 48]. The accuracy of diagnoses cannot be measured with these data. We are not able to determine the application of canalith repositioning maneuver or vestibular rehabilitation in any of the groups, nor predictors of chronic symptoms (anxiety, depression, migraine, chronic pain, obesity etc.).

This study deals exclusively with a group of actively employed individuals. The increased prevalence of dizziness/vertigo among the older population, i.e., pensioners, was not within the aim of the current study. The study was not designed to identify psycho-social predictors of sick leave and the post-hoc finding regarding education and income among women should be studied further before drawing firm conclusions.

## Conclusion

A high proportion of sick leave spells in Sweden are due to nonspecific dizziness/vertigo, highlighting the need to improve the diagnostic efforts when issuing sick leave certificates. This, together with the finding that a large proportion were on sick leave due to benign paroxysmal positional vertigo, implies that vestibular rehabilitation and repositioning maneuvers may be underutilized.

Sick leave caused by nonspecific diagnoses and BPPV were more common among women than men, and sick leave in women with high education seems independent of age with acceleration already in the 4th decade. These observations mandate further research to confirm and elucidate.

### Take Home Message

Vertigo/dizziness is a subjective feeling and therefore difficult to diagnose by the physician especially in the absence of objective clinical symptoms or findings. Attempts to use Artificial Intelligence to separate vestibular vertigo from nonspecific dizziness has failed [49]. The authors feel inclined to recommend that before issuing sick leave certificates due to R42 and H81.9, at least some kind of quantitative dizziness questionnaire and a screening instrument for psychiatric comorbidity should be used [11], together with a screening for common workplace stressors. Participation in treatment for the suspected underlying cause, such as particle repositioning maneuvers, and general vestibular rehabilitation should be given high priority [27, 50, 51].

## Data Availability

The Swedish Social Insurance Agency and Statistics Sweden regulations prohibits data sharing across borders. Aggregated data will be made available from the first author on reasonable request. Dr. Katarina Zborayova may be reached at Katarina.Zborayova@regionvasterbotten.se.

## Abbreviations

BPPV: Benign paroxysmal positional vertigo;
CSD: Chronic subjective dizziness
GP: General practitioner
ICD-10: International Statistical Classification of Diseases and Related Health Problems, version 10
ICPC: International Classification of primary care
ICVD: International Classification of vestibular disorders
LISA: Longitudinell Integrationsdatabas för Sjukförsäkrings- och Arbetsmarknadsstudier
MSCR: Medical Sickness Certificates Register
Vertigo NOS: Vertigo Not otherwise specified
PPPD: Persistent postural perceptive dizziness
PPV: Phobic postural vertigo
SSIA: Swedish Social Insurance Agency
